# A Cross-Sectional Study to Assess HIV/AIDS-Related Stigma and Its Drivers Among Dental Healthcare Providers in Islamabad, Pakistan

**DOI:** 10.7759/cureus.46769

**Published:** 2023-10-09

**Authors:** Beenish K Rana, Mariyam Sarfraz, Tahira Ezra Reza, Faran Emmanuel

**Affiliations:** 1 Global Public Health, Health Services Academy, Islamabad, PAK; 2 Epidemiology and Public Health, Centre for Global Public Health, Islamabad, PAK; 3 Epidemiology and Public Health, University of Manitoba, Winnipeg, CAN

**Keywords:** dental healthcare providers, infectious diseases, drivers of stigma, stigma in health care, hiv aids

## Abstract

Introduction

HIV-related stigma and discrimination among healthcare providers are some of the strongest obstacles to effectively responding to HIV and achieving health-related quality of life. In the Pakistani context, HIV-related discrimination has been explored mainly among people living with HIV (PLHIV), and no study has investigated HIV-related stigma from the perspective of dental healthcare providers.

Aim

This study aimed to investigate the HIV-related stigma among dental healthcare workers in Pakistan and understand the factors associated with it.

Methodology

This cross-sectional study was conducted for a period of three months (December 2021 to February 2022) among 601 consenting dental healthcare providers in all public and private dental healthcare facilities in Islamabad, Pakistan. Pretested questionnaires collected information on demographics, work-related characteristics, knowledge, attitudes, and practices regarding HIV/AIDS. HIV-related stigma was assessed through “the stigma index” developed by USAID and was measured as a continuous variable. Multivariate linear regression analysis evaluated the independent effect of different factors associated with HIV-related stigma.

Results

HIV-related stigma remains highly prevalent within both public and private dental healthcare facilities in ICT and among all cadres of dental healthcare providers. Among associated factors, misconceptions in HIV knowledge are highly significant (p < 0.001) and those with a higher score of incorrect HIV knowledge had higher levels of stigma. Healthcare providers who read any HIV-related manual or guidelines were found to be less stigmatized as compared to those who have not been exposed to any such literature (p=0.029). Dentists (p=0.04) showed higher levels of stigma as compared to dental assistants and dental hygienists, while employees of private hospitals (p=0.0) and private clinics (p=0.0) were far more stigmatized by HIV in comparison to dental healthcare providers in public hospitals.

Conclusion

This study provides the first-ever analysis of HIV-related stigma and its drivers in the dental healthcare settings in Pakistan and highlights multiple individual, clinical, and policy-level factors associated with it. In order to address this stigma, it is essential for healthcare institutions to create supportive and inclusive healthcare settings, by providing education and training to care providers in order to increase their understanding of the disease itself. In addition, healthcare institutions can take steps to ensure that their policies and practices are inclusive and non-discriminatory, such as implementing policies that prohibit discrimination based on HIV status and providing confidential care. On the other hand, care providers must work to recognize their own biases and strive to provide non-discriminatory and culturally sensitive care to all patients.

The findings of this study could be used as a baseline and insight by organizations like the National AIDS Control Program into possible targets for future exploration and interventions to effectively reduce the stigma toward PLHIV in dental healthcare settings.

## Introduction

Infection with human immunodeficiency virus type-1 (HIV) and the consequential acquired immune deficiency syndrome (AIDS) is one of the most important public health challenges in the present times having claimed approximately 34.7 million lives so far. According to the AIDS epidemic report of 2022, the global burden of HIV/AIDS was approximately 37.6 million cases, with a prevalence rate of about 476 cases per 100,000 [1]. In Pakistan, the epidemiology of HIV has drastically increased over the past few years, shifting the country’s paradigm from a low prevalence status to a highly concentrated epidemic among key populations [2], with approximately 190,000 people living with HIV [3].

HIV/AIDS has always remained a globally stigmatized disease associated with disgrace and unacceptability [4]. Over the years, while advances in treatment and better prevention strategies have led to encouraging gains worldwide and optimism in improving the lives of people living with HIV, the associated stigma and discrimination persist and continue to be a significant challenge for people living with HIV [5]. The Joint United Nations Programs on HIV/AIDS (UNAIDS) defines stigma and discrimination as a process of devaluation of people either living with or associated with HIV and AIDS; discrimination follows stigma which is the unfair and unjust treatment of an individual based on his or her real or perceived HIV status [6]. HIV-related stigma persists within all settings but has far more serious consequences when occurring in healthcare settings manifesting itself as marginalization and discrimination resulting in violation of human rights [7]. Although healthcare professionals have an ethical duty to avoid engaging in stigmatizing behaviors and a legal duty not to discriminate, HIV stigma and discrimination among healthcare workers have been extensively documented in the literature [7-9]. HIV-related stigma is shown to exist at multiple levels in the healthcare setting: individual level (e.g., personal attitudes, beliefs, and behaviors), health facility level (e.g., clinic characteristics, type, and location), and policy level (e.g., institutional policies, support, and training). Stigmatizing practices of healthcare providers include delayed treatment, refusal by healthcare professionals to perform procedures, poor service quality, lengthy tests, and negative attitudes [10-12]. Fear of acquiring infection, poor knowledge, lack of proper guidelines, traditional values and cultural norms, religious issues, and thoughts were reported as drivers behind these stigmatizing behaviors [13-18].

While dentists all over the world have shown to have differential attitudes toward treating HIV-positive individuals [7], there is a scarcity of literature that documents HIV-related stigma and its associated factors among dental healthcare providers in Pakistan. With the growing epidemic of HIV in Pakistan, it is imperative to investigate the presence of HIV-related stigma among dental healthcare workers in Pakistan and understand the factors associated with it.

## Materials and methods

An analytical cross-sectional study was conducted among the registered and practicing dental healthcare providers in Islamabad Capital Territory (ICT) Pakistan. Data were collected between December 2021 and February 2022 from registered (licensed by the Pakistan Medical & Dental Council) dentists, dental hygienists, and dental assistants (both male and female) who were currently working in public and private sector health facilities in ICT, Pakistan. 

Sample size and sampling strategy

The sample size was determined both to estimate the prevalence of stigma among dental care providers and to assess its drivers and associated factors. We added a design effect of 1.5 and increased the calculated sample size by 10%, to account for missing or incomplete data. Since our outcome variable was continuous (a score for HIV-related stigma), we calculated a sample size of 601 which can detect values of R2 ≥ 0.2 with 12 independent variables in the model with an α of 0.05 and a power of 90%.

A list of all registered dental institutions (public and private) and private dental clinics in Islamabad was obtained from the Ministry of National Health (MoNHSRC), along with a list of all healthcare providers in each institute. Sample selection was done using the multistage random sampling technique, where dental health facilities were selected in the first stage randomly, and eligible respondents were selected from each health facility in the next stage. In case more than one eligible respondent was found in a health facility, a random selection of respondents was done.

Field data collection

The data collection team comprised the principal investigator, supported by four field researchers (two males and two females) trained in dentistry and public health. The field team was further trained by the principal investigator in a three-day training on field data collection methods and research procedures. The training focused on familiarizing field researchers with the questionnaire, communication and interviewing skills, ethical considerations for research, sampling procedures, and how to enter data in the database developed for this study. Prior to commencing field data collection, the questionnaire was pretested in 10 health facilities in Rawalpindi, where 30 interviews were conducted. The pilot testing highlighted all issues related to the questionnaire which were addressed and corrected in the final research implementation.

A field team comprising a male and a female interviewer visited each health facility after a prior appointment was taken telephonically. Within each health facility, one dentist, one dental hygienist, and one dental assistant were interviewed. When more than one eligible participant was available, the respondent’s selection was done randomly (i.e., one dentist, one hygienist, and one assistant). Respondents were briefed about the study and written consent was taken prior to the interview. Data were collected in a face-to-face interview which lasted for nearly 20 minutes. Data collection was completed in a period of two months and 200 health facilities (three public hospitals, three rural health centers, eight private hospitals, and 186 private clinics) were included in the study. Data were collected using a pretested questionnaire, which comprised four sections. These included variables related to sociodemographic factors, occupational characteristics, knowledge, practices, and attitudes related to HIV. A globally standardized scale developed by USAID-funded Health Policy Projects (Cronbach’s alpha of 0.95) was used to access stigma, which was evaluated by ranking statements on a four-point Likert scale.

Data management and analysis

Real-time data were collected using hand-held tablets and data were directly entered into the database developed in the Kobo toolbox [19]. All data files were uploaded to the database after individual forms were checked and validated at the end of each day for their completeness, missing values, consistency, and meaningfulness of the information collected. Data were analyzed using IBM SPSS Statistics for Windows, Version 26 (Released 2019; IBM Corp., Armonk, New York, United States). The database was password-protected, and data access was limited to the principal investigator only.

The dependent variable for the current analyses was “stigma score associated with HIV/AIDS” which was measured as a continuous variable. Each item in the stigma scale had four probable answers with values from one to four, with one representing “strongly agree” and four representing “strongly disagree”. One out of the 13 scale items were positively formulated while the remaining 12 were stated inversely with regard to what is considered as more stigmatized. The codes of 12 questions were reversed such that the higher values are indicative of more stigma. Sums of item scores were used to calculate the dependent variable i.e., “stigma score” out of a total score of 52 with a higher number indicating more stigma.

Factors associated with the outcome were divided into three broad categories: Individual-level measures including sociodemographic characteristics (age, sex, marital status, income, and highest level of education), work history (years in the healthcare profession, years in current job, current job category, knowledge, attitudes and practices of HIV, infection control, HIV testing, mode of spread, and prevention). Clinic-level measures included access to personal protective equipment at the facility and practices followed at their facility. Policy-level measures included the presence of procedural protocols, standardized operational guidelines, and their enforcement.

The HIV knowledge score was a composite variable obtained by summation of the correct responses to six knowledge questions. The score for HIV misconceptions was calculated using eight different questions; higher scores indicated poor knowledge. Likewise, the score for incorrect practices comprised responses to four questions and a higher score indicated more incorrect practices. 

Descriptive analysis included calculating frequencies and percentages for various demographic and work-related characteristics as well as respondent’s knowledge, attitude, and practices regarding HIV/AIDS. To understand the various factors associated with stigma, they were evaluated using multiple linear regression models. The “stigma score” calculated for each study participant was regressed against each of the possible and plausible determinants of stigma to look for statistically significant associations through univariate analysis using linear regression analysis. This was followed by multivariate linear regression analysis to evaluate the independent effect of each of the variables found to be statistically significant in univariate analysis. All sociodemographic factors, individual-level factors, and clinical factors which were found significant in univariate analysis were analyzed using different regression models, and the best-fit model (which has the least sum of squares, best p-value) was selected.

## Results

Sociodemographic and clinical characteristics

A total of 601 dental healthcare providers from 200 dental facilities were included in the study (n=601) as shown in Table 1. Three participants refused to participate (in private hospitals) and another staff member of the same cadre was selected randomly to replace the non-participating individual. Among the respondents, 355 were males (59.1%) and 246 were females (40.9 %). More than one-third of the respondents were within 26 to 30 years, with a mean age of 31.8 ± 7.8 years of the sample population. The median income was 40,000 PKR. Since we included all dental service providers in this study, our sample included 64.8% (n=388) dentists, and 15.0% (n=90) dental assistants while the remaining 20.5% (n=123) were dental hygienists. Approximately 73.4% (n=441) of the respondents had less than 10 years of professional experience. Sixty-nine percent of the respondents were working in private clinics, 24.8% were working in private hospitals, and the remaining 5.8% (n=35) were employed in public hospitals. Only 10.8% (n=65) were trained outside of Pakistan, while 9.8% (n=59) also had international work experience. The cost of a root canal was used as a proxy indicator to measure the treatment affordability of these dental facilities. Nearly 40% (n=243) of the facilities were economical, 31.1% (n=187) were moderately expensive, and 28.5% (n=171) facilities were found to be very expensive. 

**Table 1:** Sociodemographic and work characteristics of dental care providers

Variable	N = 601	% Age
Age (in years)		
Upto 25 years	112	18.6
26 to 30 years	214	35.6
31 to 35 years	130	21.6
More than 35 years	145	24.1
Mean ± SD	31.8 ± 7.8	
Gender		
Male	355	59.1
Female	246	40.9
Income in PKR (Median: 40000)		
Upto 20,000 PKR	144	24.0
21,000 to 30,000 PKR	107	17.8
31,000 to 60,000 PKR	135	22.5
61,000 to 200,000 PKR	113	18.8
More than 200,000 PKR	102	17.0
Professional experience in years		
Upto 5 years	272	45.3
6 to 9 years	169	28.1
10 years and more	160	26.6
Job title		
Dentist	388	64.8
Dental assistant	90	15.0
Dental hygienist	123	20.5
Type of health facility		
Private hospital	149	24.8
Public hospital	35	5.8
Private clinic	417	69.4
Had received training outside of Pakistan	65	10.8
Had experience of working outside of Pakistan	59	9.8
Have read published guidelines about HIV/AIDS	95	15.8
Affordability (Charges of root canal in PKR)		
Reasonably affordable ( less than 10,000 PKR)	243	40.4
Moderately expensive (10,001 to 16,000 PKR)	187	31.1
Expensive (more than 16,000 PKR )	171	28.5

Knowledge, attitudes, and practices of dental healthcare providers

Table 2 presents the HIV-related knowledge, attitudes, and practices of dental care providers. All study participants knew of the disease although less than 20% had attended any formal training (17.1%, n=103) or had read any formal guidelines (15.8%, n=95) about the disease. Regarding modes of transmission, “sexual intercourse” as a route of spread had the highest knowledge (98.7%) followed by the knowledge of “spread through sharp instruments” (97.2%), through blood transfusion (91.8%), vertical transmission (88.5%) and through saliva (48.9%). Misconceptions about the routes of disease e.g., by “kissing/hugging and touching (38.3%), eating and drinking with people with HIV (PLHIV) (34.4%) “staying away from patients (52%), and “refraining from sex (47.6%) were also noted. Among all the participants, 91.5% (n=550) considered themselves at the risk of acquiring HIV/AIDS; 95% (n=579) thought that despite clinical precaution there is a risk of acquiring HIV, and 89% (n=534) of the respondents were hesitant to treat HIV patients. When asked about practices among all participants, 98% (n=591) responded that they would use special infection control measures with HIV patients and 97.5% (n=586) reported that they would skip one or more steps of treatment while providing dental care to HIV patients. Ninety percent (n=541) of the respondents claimed that they will avoid physical contact with PLHIV and 91.2% (n=548) will keep HIV patients separate from the other patients. 

**Table 2:** Knowledge, attitude, and practices of dental care providers regarding HIV/AIDS PLHIV: People living with HIV

Variable	n	%
Heard about HIV/AIDS	601	100
Attended training/ seminar on HIV	103	17.1
Read any published HIV manuals/guidelines	95	15.8
HIV can be transmitted through		
Sexual intercourse	593	98.7
Sharp instruments	584	97.2
Mother to child	532	88.5
Blood transfusion	552	91.8
Eating and drinking with the patients	207	34.4
Kissing/ hugging and touching	230	38.3
Saliva	294	48.9
HIV can be prevented through		
Safe sex	488	81.2
Safe blood transfusion	555	92.3
Infection prevention protocols	512	85.2
Sterilized instruments	571	95.0
Refraining from sex	286	47.6
Staying away from patients	313	52.1
Ever treated a patient with HIV	351	58.4
Provision of treatment with the same protocol (won't skip any steps of treatment)	15	2.5
Use of special infection-control measures with PLHIV which is not used with other patients	591	98.3
Avoiding physical contact with a patient of HIV/AIDS	541	90.1
Keeping a person with HIV/AIDS separate from other patients and staff	548	91.2
Ever treated a patient with HIV	351	58.4

Prevalence of stigma

As shown in Table 3, a significant number of respondents hold stigmatizing attitudes toward people living with HIV; 61.8% (n=371) of the respondents agreed that people living with HIV should be ashamed of themselves, and 96% (n=577) of respondents believed that people living with HIV don't care if they infect others. Additionally, 76.6% (n=461) of the respondents believed that PLHIV have multiple sexual partners while 69.4% (n=417) were of the view that HIV is a punishment for bad behavior. If given a choice, 55.2% (n=332) of the respondents will not prefer to treat men who have sex with men while 57.4% (n=345) will not prefer to provide treatment to female sex workers. Only 29.1% (n=175) of the healthcare workers believed that people with AIDS should be treated similarly to people with other illnesses, and only 25.6 % (n=154) believed that they should be allowed to fully participate in social events in the community and work with others. 

**Table 3:** Prevalence of stigma of HIV/AIDS among dental healthcare providers PLHIV: People living with HIV; MSM: men who have sex with men; FSW: female sex worker

	Variable	Strongly Agree	Agree	Disagree	Strongly Disagree
		%	%	%	%
1	PLHIV should be ashamed of themselves	15.5	46.3	21.6	16.6
2	PLHIV don’t care if they infect other people	61.2	34.8	4.0	0.0
3	PLHIV have had many sexual partners.	45.9	30.8	16.6	6.7
4	HIV is punishment for bad behavior	20.3	49.1	18.3	12.3
5	Would prefer not to provide services to MSM	5.8	49.4	18.5	26.3
6	Would prefer not to provide services to FSW	6.3	51.1	17.0	25.6
7	In the past 12 months, I have observed healthcare workers talking badly for PLHIV	1.0	13.0	42.1	43.9
8	In the past 12 months, I have observed healthcare workers providing poorer quality of care to a patient living with PLHIV	44.1	43.9	10.8	1.2
9	In the past 12 months, I have observed healthcare workers unwilling to take care of PLHIV	5.2	44.6	28.0	22.3
10	How worried are you about friends and family avoiding you because you care for patients living with HIV?	13.6	57.9	26.6	1.8
11	How worried are you about colleagues avoiding you because of your work caring for patients living with HIV?	12.1	43.1	41.1	3.7
12	PLHIV should be treated similarly by healthcare professionals as people with other illnesses	11.8	17.3	47.4	23.5
13	PLHIV should be allowed to fully participate in social events in the community and should be allowed to work with other people	7.0	18.6	61.2	13.1

Factors associated with HIV-related stigma

Table 4 shows the results of the univariate analysis using linear regression. The “stigma score” calculated for each study participant was regressed against each of the possible and plausible determinants of stigma to look for statistically significant associations. All factors found to be significantly associated with HIV stigma in univariate analysis were entered into a multivariate linear regression model to evaluate the independent effect of each of these variables.

**Table 4:** Univariate analysis

Variable	B	Std.	t	Sig
Age	-.068	.03	-2.15	0.031
Gender: ref (female)	-0.62	.49	0.13	1.041
Monthly income	-0.000004749	.00	-3.66	0.000
Years of professional experience	-.118	.04	-2.86	0.004
HIV Knowledge misconception	.652	.10	6.17	0.000
Incorrect practices	3.97	.38	10.4	0.000
Have read published HIV guidelines	3.37	.65	5.14	0.000
Received foreign training	1.62	.78	2.06	0.039
Specialty: ref (dental hygienist)				
Dentist	1.58	.61	2.60	0.001
Dental assistant	2.28	.69	3.30	0.011
Type of health facility: ref (Pub.Hos)				
Private hospital	11.1	1.0	10.92	0.000
Private clinic	6.90	.95	7.23	0.000
Type of health facility w.r.t affordability				
Expensive	2.95	.58	10.92	0.000
Moderate	1.56	.57	7.23	0.000

Table 5 presents the results of the final regression model. Misconceptions in HIV knowledge are highly significant (p < 0.001) and those with a higher score of incorrect HIV knowledge had higher levels of stigma. Healthcare providers who read any HIV-related manual or guidelines were found to be less stigmatized as compared to those who haven’t been exposed to any such literature (p=0.029). Dentists (p=0.04) showed higher levels of stigma as compared to dental assistants and dental hygienists, while employees of private hospitals (p=0.0) and private clinics (p=0.0) were far more stigmatized by HIV in comparison to dental healthcare providers in public hospitals. 

**Table 5:** Multivariable analysis

	Coefficient	Std.	t	Sig	(95% confidence interval)	
					Lower	upper
HIV Knowledge misconception	0.42	.099	4.30	0.00	.23	.62
Have read published guidelines (ref=read)	1.40	.640	2.19	0.02	.14	2.66
Specialty (ref dental hygienist)						
Dentist	1.19	.586	2.03	0.04	2.3	.04
Dental assistant	0.59	.636	0.94	0.34	-.65	1.84
Type of health facility (ref public hospital						
Private hospital	8.59	1.08	7.91	0.00	6.46	10.73
Private clinic	5.12	1.01	5.05	0.00	3.12	7.11
Type of health facility w.r.t affordability (ref economical)						
Private hospital	1.04	.540	1.92	0.05	-.02	2.10
Private clinic	1.34	.561	2.40	0.01	.24	2.45

## Discussion

Although stigma and discrimination associated with HIV/AIDS have been comprehensively researched globally, developing countries like Pakistan lack well-designed exploratory studies to document stigma among healthcare providers, especially dental care providers. This study thus fills this knowledge gap and is the first to report the presence of HIV/AIDS-related stigma among dental care providers in Pakistan, highlighting some of the associated factors as well.

Results show that HIV-related stigma remains highly prevalent within all types of dental care facilities in ICT, among all various cadres of providers. The results are consistent with other studies that have found similar levels of stigma and discrimination among healthcare workers against PLHIV [7,8]; more than half of the study participants believed that PLHIV should be ashamed of themselves. Similarly, the belief that HIV is a punishment for bad behavior perpetuates the myth that HIV/AIDS is a result of moral failing rather than a disease that can affect anyone [20,21]. These behaviors not only impact the quality of care and treatment provided by the healthcare workers, but also lead to self-stigmatization among PLHIV and can have negative effects on their mental health, treatment adherence, and overall quality of life. To avoid this behavior from healthcare providers, PLHIV are compelled to provide false information or conceal their HIV status, potentially resulting in spread of infection. Our findings are consistent with similar studies, [18,22,23] although more stigma is seen in Pakistan as compared to studies conducted in developed nations [14,24,25]. Another concern highlighted through this research is the stigmatizing and discriminating attitude of dental care providers toward key populations, which is also documented extensively in the literature [26]. More than half of the dental care providers showed reluctance to treat men who have sex with men and female sex workers. This reluctance of providers to treat these patients fuels the existing health disparities and exacerbates inequalities in healthcare access and outcomes, particularly related to the management of sexually transmitted infections like HIV [27].

In addition, this study was also able to find specific individual-level, clinic-level, and policy-level factors that influence these behaviors and could be targeted for stigma-reduction interventions. Knowledge misconceptions about HIV rather than having correct knowledge of HIV stands out to be one of the key determinants of stigma among dental care providers as shown by research conducted previously [16,28]. Incorrect knowledge and misconceptions about HIV transmission lead to unfounded fears and anxieties among healthcare providers, which may result in a heightened sense of stigma, as providers perceive PLHIV as a potential source of harm or danger. This fear can lead to avoidance behaviors toward patients and exhibit discriminatory attitudes, resulting in the patient feeling judged, devalued, or marginalized. Among occupational characteristics of dental care providers, higher levels of stigma were seen among private providers as compared to public facilities, which is consistent with previous studies [18,29]. In Pakistan, private dental hospitals and clinics usually cater for the elite class and have the choice to select patients as per their desire. Public clinics and hospitals are exposed to all different social and economic backgrounds and don’t have the choice to refuse anyone, which indirectly develops a less stigmatizing behavior. Lack of specific training on how to deal with PLHIV may result in dental healthcare providers relying on stereotypes and assumptions about PLHIV. In Pakistan, the usual medical curriculum does not incorporate any such training and thus there exist many stereotypes among healthcare providers that can lead to discriminatory behaviors, such as treating patients differently or providing suboptimal care [30]. Our results show that only 17.1 % of the participants received any form of training on HIV which is far lesser than in many neighboring countries [29]. Further to this, none of the study’s participants was aware of any institutional policies or guidelines that could enrich their knowledge of HIV AIDS and provide them with guidance on how to deal with HIV AIDS patients. This is rather disappointing as in comparison to similar other countries a large number (72.4% to 97%) of the dental healthcare providers reported having policies at their workplace to protect PLHIV from stigma [9,11,24]. Although previous research has suggested that healthcare providers have supported the need for training on HIV [18], since Pakistan is still considered to be a low HIV prevalence country, the subject hasn’t received due attention from the public health authorities and policymakers. Consistent with previous research, dentists showed higher levels of stigma in comparison to dental hygienists and dental assistants. It is an established fact that healthcare providers who have higher degrees have more detrimental attitudes and show more prejudice due to a wider gap in social status between them and PLHIV [18,29]. Since dentists have direct contact and closer patient interactions compared to dental assistants and dental hygienists, this close proximity during dental procedures might lead to heightened concerns or fears about potential exposure to HIV. These concerns can manifest as stigma if dentists hold misconceptions or stigmatizing beliefs about HIV transmission.

Stigma and discrimination have prompted serious consequences resulting in denial of testing and access to care in PLHIV and also increase the likelihood of patients hiding their HIV status resulting in the spread of disease. Our study provides evidence that there is a high level of stigma and discrimination present within the dental healthcare providers and healthcare system against HIV/AIDS in Pakistan. One of the major strengths of this study is the representativeness of all dental healthcare facilities in ICT, including private clinics, private dental hospitals, and dental departments of tertiary care hospitals as well as a representation of all different types of dental care providers in the sample. The use of a standardized stigma scale and multivariate analysis to highlight determinants of stigma are additional strengths worth mentioning. One of the key limitations is collecting data from only one city; however, the results might not be very different in other geographical settings in Pakistan too, and further research could be conducted to build these findings.

## Conclusions

This study provides the first-ever analysis of HIV-related stigma and its drivers in the dental healthcare settings in Pakistan and highlights multiple individual-, clinical-, and policy-level factors associated with it. In order to address this stigma, it is essential for healthcare institutions to create supportive and inclusive healthcare settings, by providing education and training to care providers in order to increase their understanding of the disease itself. In addition, healthcare institutions can take steps to ensure that their policies and practices are inclusive and non-discriminatory, such as implementing policies that prohibit discrimination based on HIV status and providing confidential care. On the other hand, care providers must work to recognize their own biases and strive to provide non-discriminatory and culturally sensitive care to all patients.

The findings of this study could be used as a baseline and insight by the National Aids Control Program and Ministry of Health into possible targets for future exploration and interventions to effectively reduce the stigma toward PLHIV in dental healthcare settings. Addressing HIV-related stigma among healthcare workers is essential to ensure that all patients receive the care they need and deserve to improve health outcomes and reduce health disparities for marginalized groups in Pakistan.
